# Spinal Schwannoma With Intrathoracic Extension: Successful En Bloc Resection via Uniportal Video-Assisted Thoracoscopic Surgery (VATS)

**DOI:** 10.7759/cureus.89027

**Published:** 2025-07-30

**Authors:** Len En Yean, Sivakumar Krishnasamy, Nicolas Ong Zhe Sheng, Ooi Jia Ying, Mun Kein Seong

**Affiliations:** 1 Cardiothoracic Unit, Department of Surgery, Universiti Malaya Medical Centre, Petaling Jaya, MYS; 2 Faculty of Medicine, MAHSA (Malaysian Allied Health Sciences Academy) University, Jenjarom, MYS; 3 Department of Pathology, Faculty of Medicine, Universiti Malaya, Kuala Lumpur, MYS

**Keywords:** case report, intrathoracic extension, multidisciplinary surgical approach, spinal schwannomas, uniportal video-assisted thoracoscopic surgery

## Abstract

Schwannomas are benign, well-encapsulated tumors arising from Schwann cells, which are responsible for the myelination of peripheral and central nerves. This report highlights a rare incidence of a T4 thoracic schwannoma with right paraspinal extension into the thoracic cavity. A 77-year-old woman presented with bilateral lower limb weakness; MRI revealed a T3-T5 intradural extramedullary lesion extending from the spinal canal into the right posterior mediastinum. A multidisciplinary surgical approach was employed: the neurosurgical team first performed a posterior laminectomy to resect the intraspinal component, followed by en bloc resection of the intrathoracic tumor via uniportal video-assisted thoracoscopic surgery (VATS) by the cardiothoracic team. This combined approach enabled complete tumor excision and favorable neurological recovery, highlighting the importance of coordinated multidisciplinary management in complex thoracic schwannomas with intraspinal extension.

## Introduction

Schwannomas, also known as neurinomas or neurilemmomas, are benign tumors that arise from Schwann cells responsible for myelinating both central and peripheral nerves [1]. These tumors are generally slow-growing, encapsulated, and present as well-defined, eccentrically located masses [2]. A histopathological analysis can reveal densely cellular Antoni A areas with palisading spindle cells or looser Antoni B areas with edematous stroma and fibrillar collagen [3]. Schwannomas often have prominent blood vessels encased in dense fibrous tissue. Typically arising from cranial or spinal nerves, about 45% occur in the head and neck, though thoracic nerves may also be affected [4].

The World Health Organization (WHO) classifies schwannomas as benign grade I tumors, with fewer than 1% progressing to malignancy. Although the mechanisms underlying their development are not entirely understood, these tumors are commonly associated with neurocutaneous disorders like neurofibromatosis type 1 (NF1) and type 2 (NF2). NF2 gene mutations result in merlin protein loss, a key factor in vestibular schwannoma formation. Merlin inactivation, via chromosome 22 mutations or other factors, drives schwannoma development [5].

Spinal schwannomas comprise approximately 25%-30% of all spinal tumors [6]. Many individuals with schwannomas remain asymptomatic; however, when symptoms do occur, they can vary based on the tumor's size and location. This condition occurs in 4.4 to 5.23 adults and 0.44 children per 100,000 annually [7].

Schwannomas may present as painless lumps, localized discomfort, or symptoms of nerve compression, including sensory disturbances, muscle weakness, reflex changes, or bladder and bowel dysfunction. MRI is the primary diagnostic tool for thoracic schwannomas, with computed tomography, X-rays, biopsies, and immunohistochemistry as additional options [8]. Nerve conduction studies and electromyography may aid in evaluating peripheral nerve function in cases with neurological deficits. Schwannoma management depends on location, size, symptoms, and malignancy. Surgical resection is the primary treatment, while radiosurgery (e.g., Gamma Knife) is an option for small or inoperable tumors.

This report presents a case of thoracic schwannoma successfully managed through a collaborative approach by both cardiothoracic and neurosurgical teams, providing valuable insights into the multidisciplinary treatment approach for this uncommon condition.

## Case presentation

A 77-year-old female patient with a known diagnosis of T3-T5 spinal schwannoma, extending into the right paraspinal and pleural region, was referred to the cardiothoracic team by the neurosurgical clinic for a collaborative surgical approach. The patient initially presented with bilateral lower limb weakness, first noted in July 2024. The symptoms began with left lower limb weakness, progressively involving the right lower limb within a week. She described a sensation of heaviness and weakness, necessitating the use of a wheelchair, although she could still ambulate short distances at home with the assistance of a walking frame. Additionally, the patient reported progressive numbness over the body and lower limbs, with reduced sensation beginning two weeks prior to presentation. The numbness initially involved the abdominal region and subsequently spread to both the lower body and lower limbs. Notably, the patient denied any bowel/bladder dysfunction, radicular pain, or respiratory symptoms such as shortness of breath or pleuritic chest pain.

The patient had previously undergone a left mastectomy in 2017 for stage 1 breast carcinoma in Gleneagles Hospital, Kuala Lumpur, Malaysia. Her postoperative course was uneventful, and she did not require adjuvant chemotherapy or radiotherapy. Since then, she has been under regular surveillance follow-up at the same hospital. However, routine surveillance in 2022 revealed a spinal shadow, leading to an MRI that confirmed the presence of a vertebral tumor. A biopsy was subsequently performed, and a histopathological examination confirmed the diagnosis of a benign schwannoma. Despite this, the patient remained asymptomatic until July 2024, when she developed the aforementioned neurological deficits, leading to her referral from Gleneagles Hospital to the University of Malaya Specialist Centre (UMSC), Kuala Lumpur.

At the UMSC neurosurgical clinic, the patient’s neurological examination demonstrated that she was alert and fully oriented with a Glasgow Coma Scale (GCS) score of E4V5M6. Neurological assessment showed bilateral lower limb hypertonia with muscle strength graded 3/5 in the right lower limb for L2, L3, L4, and S1 and 4/5 for L3 and L5. Hyperreflexia was noted at the knees and ankles bilaterally, with a positive Babinski sign in both lower extremities, indicating an upper motor neuron involvement. Sensory examination revealed reduced sensation below the T6 dermatome.

**Figure 1:**
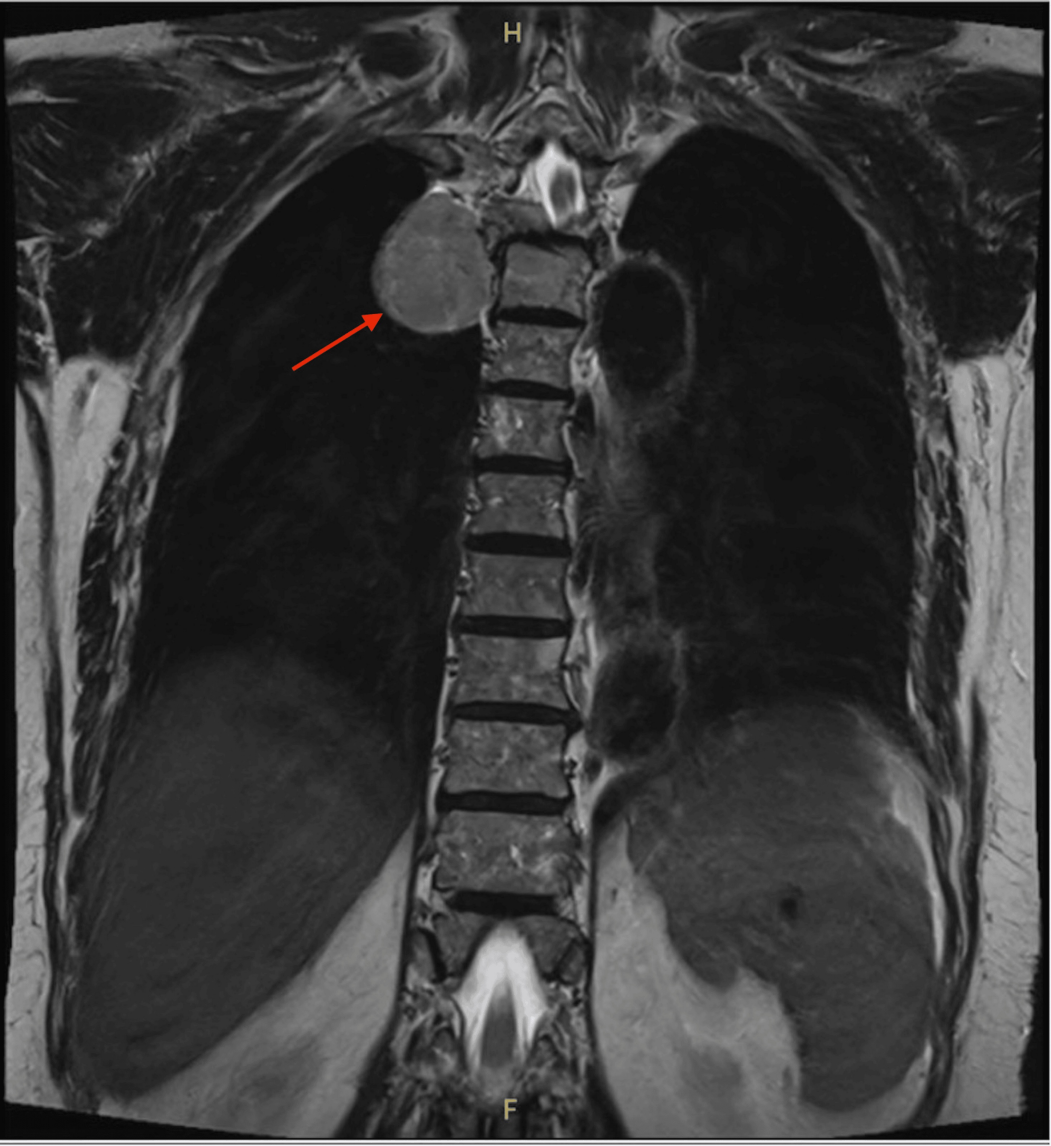
Coronal view of the thoracic MRI The red arrow shows a well-defined lobulated right-sided paraspinal mass at the thoracic vertebrae 3 to 5 (T3-T5) level, measuring 3.8 x 5.6 x 4.0 cm, with extension into the right side of the posterior mediastinum region in the thoracic cavity.

A repeat MRI requested by the neurosurgical team demonstrated an intradural extramedullary (IDEM) lesion at the T3-T5 levels with a dumbbell-shaped extension into the right paraspinal region, measuring approximately 3.8 x 5.6 x 4.0 cm (Figure 1). This prompted a referral to the cardiothoracic team to assess the tumor’s mediastinal involvement and facilitate multidisciplinary surgical planning.

To further delineate the extent of bony involvement, contrast-enhanced CT of the thoracic spine was subsequently performed. It confirmed a well-defined, lobulated paraspinal mass at the same levels with extension into the right posterior mediastinum. The lesion exhibited mild homogeneous enhancement without calcifications. Evidence of bone remodelling and erosion was observed, involving the right lamina and transverse process of T3, as well as the vertebral body, spinous process, right transverse process, and pedicle of T4. No significant pleural effusion or mediastinal lymphadenopathy was identified.

Additionally, biapical fibrosis was noted in the lungs, and stable hypodense liver lesions were identified at segment 7, measuring 1.1 x 0.9 cm, likely representing benign cysts. The radiological impression confirmed a T3-T5 spinal schwannoma with bony remodeling and thoracic extension. Although the bi-apical fibrosis and stable hypodense liver lesions were incidental findings, follow-up imaging may be warranted to monitor for any progression or underlying pathology.

Given the tumor's extension into the thoracic cavity, a multidisciplinary surgical intervention was planned, involving both the neurosurgical and cardiothoracic teams to ensure complete resection. The neurosurgical team performed the initial excision of the tumor's spinal component, followed by the cardiothoracic team addressing the intrathoracic extension at the T4 level.

**Figure 2:**
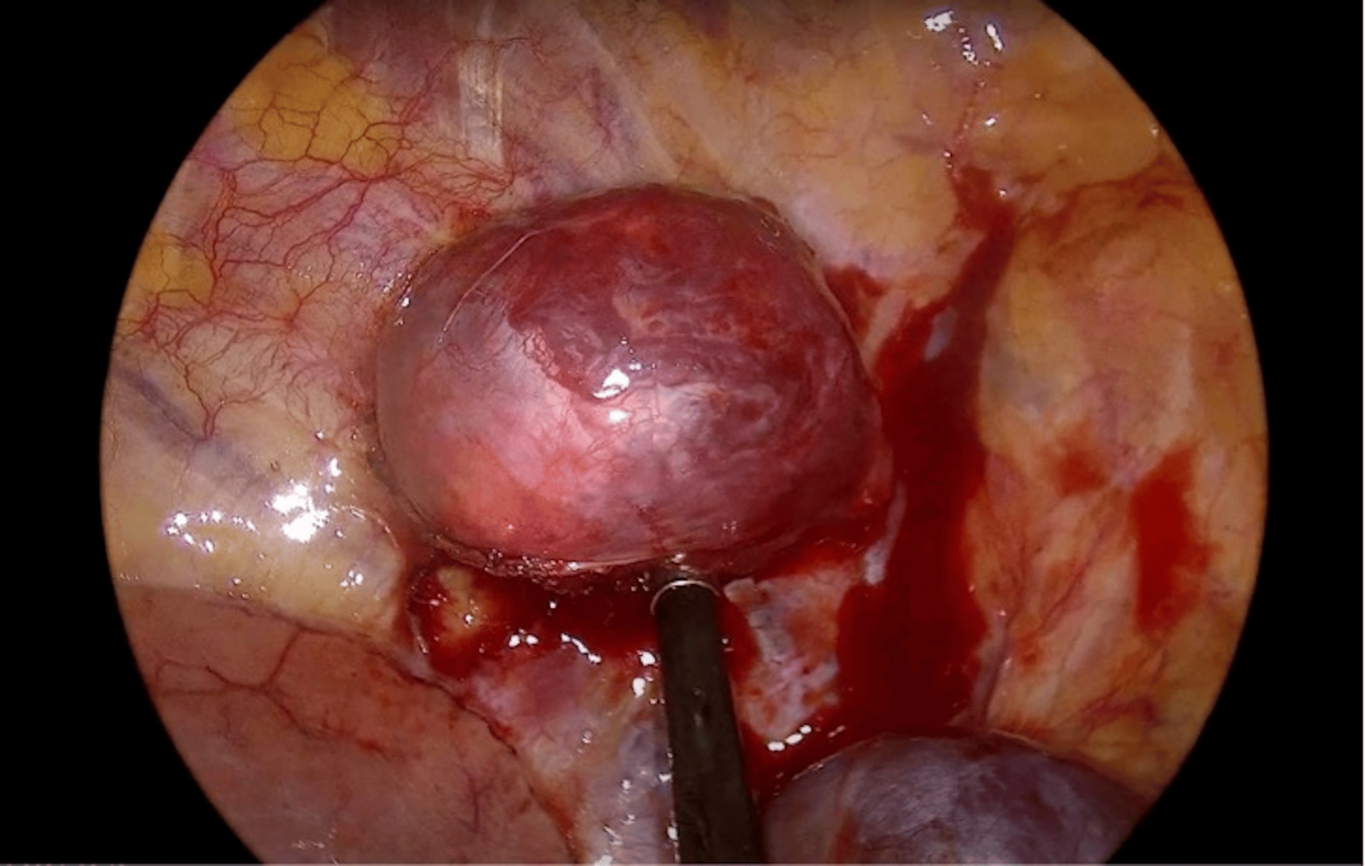
A 5 x 6 cm schwannoma, encapsulated and vascularized, located in the posterior thoracic region

Upon entering the thoracic cavity via right-sided uniportal video-assisted thoracoscopic surgery (VATS) over the fourth intercostal space at the anterior axillary line, a well-encapsulated, vascularised schwannoma, measuring 5 x 6 cm, was identified in the posterior mediastinal region (Figure 2). The tumor was noted to have breached the posterior mediastinal pleura with extension from the spinal canal. A meticulous resection was performed, achieving complete en bloc excision of the tumor.

**Figure 3:**
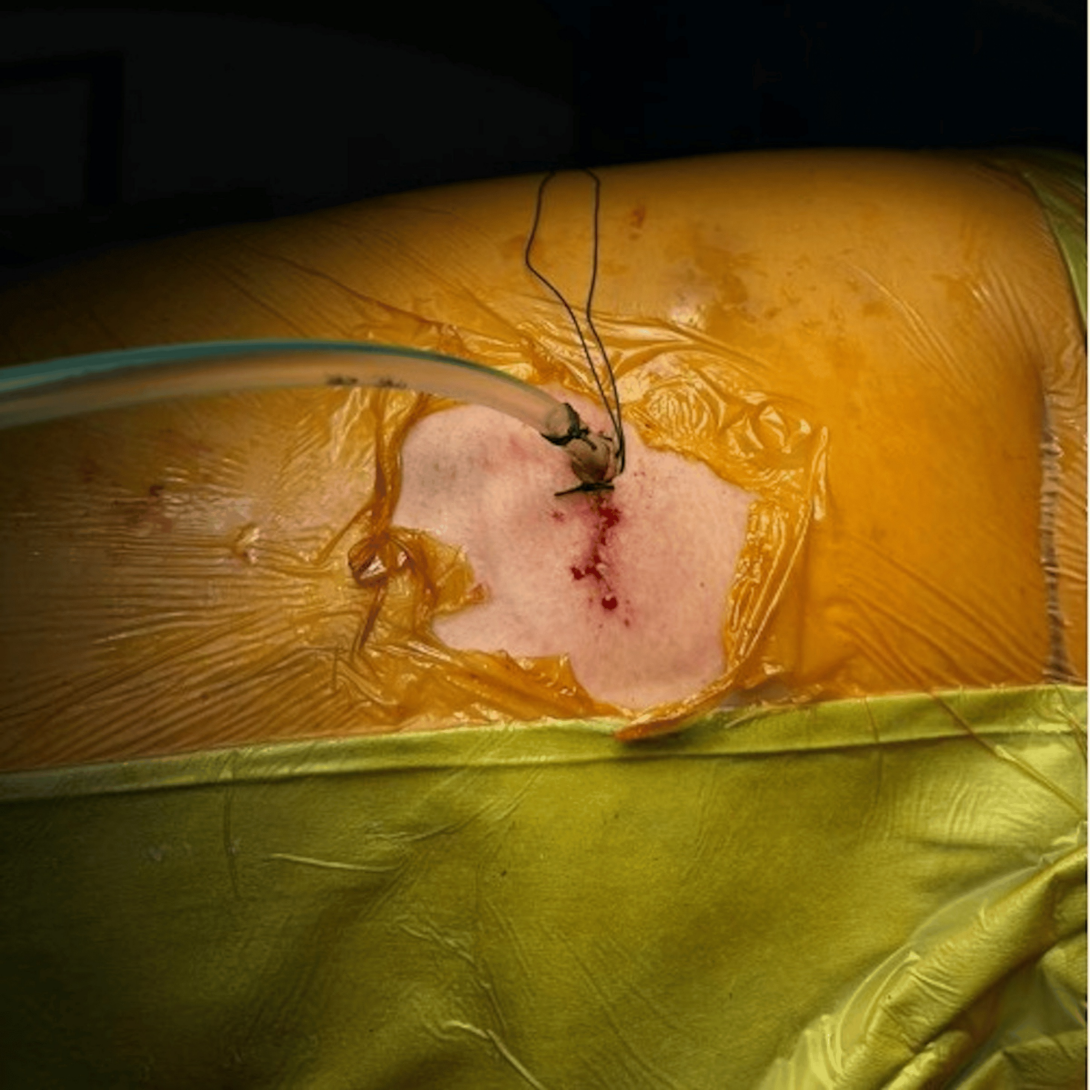
Postoperative video-assisted thoracoscopic surgery (VATS) incision site and the placement of a right pleural chest drain. A 2- to 3-cm surgical wound is visible over the fourth intercostal space at the anterior axillary line

A thorough inspection of the entire right hemithorax was conducted, confirming the absence of residual tumor tissue. Hemostasis was achieved without complications, and the tumor was excised en bloc. A right pleural chest drain was placed intraoperatively (Figure 3). The resected tumor, along with spinal and thoracic margins, was sent for histopathological examination (HPE) to assess the nature and extent of the lesion.

Postoperatively, the patient was extubated and admitted to the cardiothoracic intensive care unit (CICU) for close monitoring.

**Figure 4:**
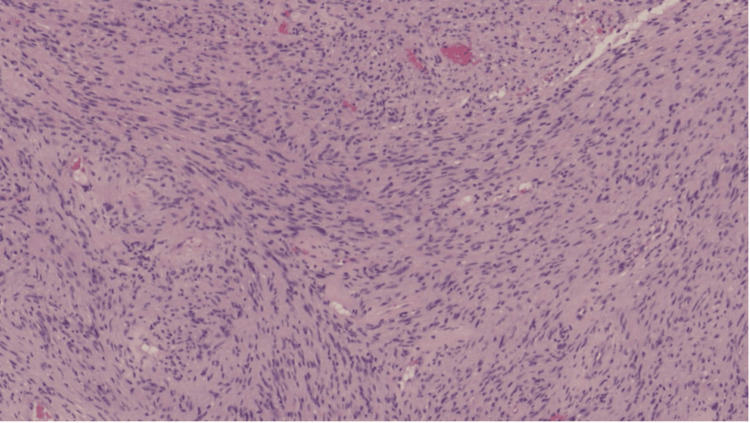
The thoracic mass with spindle cells arranged in an Antoni A pattern, with prominent nuclear palisading (Verocay bodies) Hematoxylin & eosin stain, original magnification x4 objective; scale bar = 100 µm

**Figure 5:**
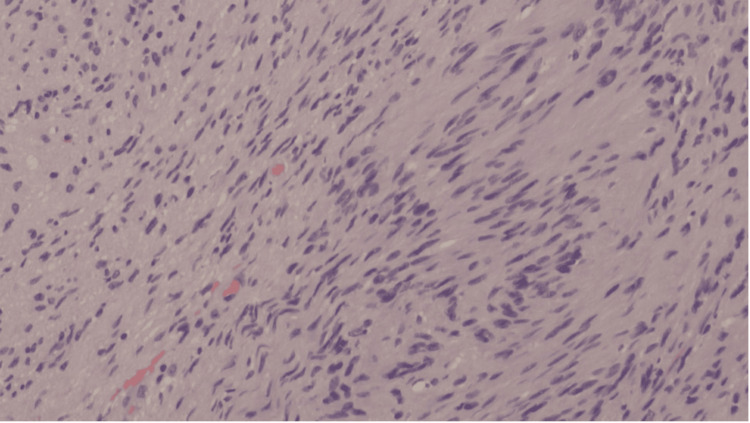
A higher magnification showing distinctive nuclear palisades Hematoxylin & eosin stain, original magnification x10 objective; scale bar = 100 µm

**Figure 6:**
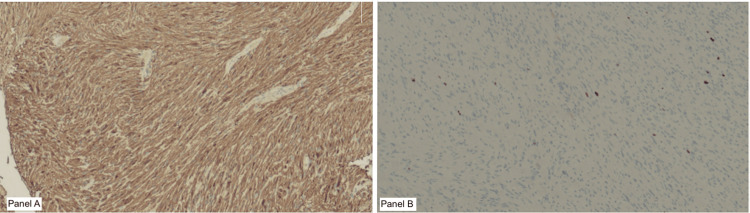
Immunohistochemistry showing strong diffuse S100 positivity (Panel A) and low Ki-67 proliferative index (Panel B) S100 and Ki-67 antibodies, original magnification x4 objective; scale bar = 100 µm

**Figure 7:**
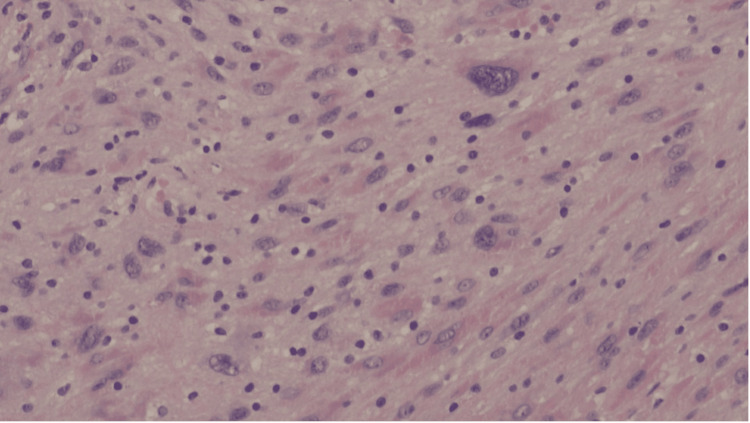
A higher magnification showing degenerative “ancient” features of pleomorphic nuclei and increased background inflammation Hematoxylin & eosin stain, original magnification x10 objective; scale bar = 100 µm

Histopathological microscopy analysis showed a well-circumscribed, unencapsulated tumor composed of spindle cells with slender, ovoid, vesicular to hyperchromatic nuclei. The tumor was characterised by the presence of cellular Antoni A areas and less cellular, oedematous Antoni B regions (Figure 4). Compact nuclear palisading was evident, forming Verocay bodies, while focal cystic degeneration was noted (Figure 5). Despite the presence of nuclear atypia, there was no evidence of mitosis or necrosis. Inflammatory infiltrates were observed, with pockets of foamy and hemosiderin-laden macrophages. Immunohistochemical analysis revealed diffuse positivity for S100, with a Ki-67 proliferative index of less than 1% in the less cellular regions, but approximately 5% in the more cellular areas (Figure 6). The overall findings were those of a benign schwannoma with a cellular pattern and degenerative (ancient) features (Figure 7).

**Figure 8:**
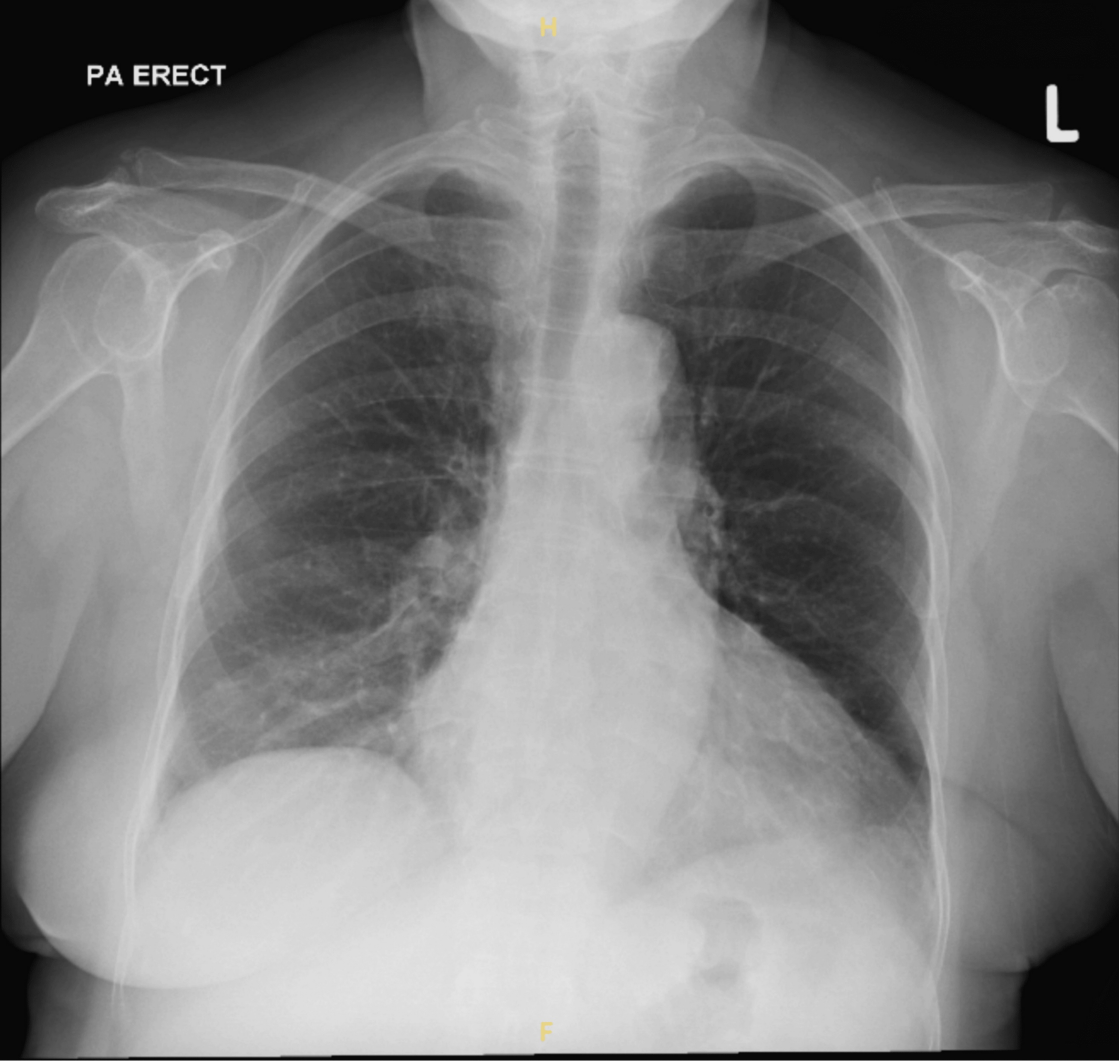
A follow-up chest radiograph at two weeks postoperatively showing no residual or recurrent mediastinal mass

At the follow-up appointment two weeks later in the surgical clinic, the patient's condition had significantly improved. Her lower limb power was measured at 4+, and she was able to ambulate independently without the need for walking aids. A follow-up chest radiograph was also performed and showed no evidence of residual or recurrent disease (Figure 8).

## Discussion

Schwannomas are defined as benign tumors that originate from Schwann cells. They can originate from myelinated or peripheral nerves, which have Schwann cells. The WHO classifies them as grade 1 benign tumors. Schwannomas in general are solitary in 90% of cases, with multiple occurrences in a patient raising suspicion of syndromic associations such as neurofibromatosis type 2, Carney complex, and schwannomatosis. They are mostly solid or heterogeneously solid tumors and rarely cystic [9]. However, in this case, the schwannoma was cystic and an intradural extramedullary lesion at the T3-T5 level of the spinal canal.

Schwannomas of the spinal canal comprise approximately 25% of all spinal canal tumors and are the second most common type of IDEM tumors following spinal meningiomas. They are less commonly seen in the thoracic and cervical regions. To the best of our knowledge, only three cases of totally cystic schwannomas of the thoracic region have been reported in the English literature [9-11]. Spinal schwannomas are most frequently observed in the lumbar region [12,13].

Despite their rarity, IDEM cystic schwannomas should be considered in the differential diagnosis of spinal cystic lesions, particularly within the thoracic region. These benign tumors arise from Schwann cells of the nerve sheath and account for approximately 8% of primary intracranial and 29% of primary spinal tumors. Together with meningiomas, schwannomas represent the most common type of IDEM tumors in the thoracic spine [14]. This case highlights the critical importance of early diagnosis and coordinated multidisciplinary surgical management in achieving favourable outcomes in patients with such uncommon and anatomically complex lesions.

Clinical signs and symptoms of spinal schwannomas are absent until the tumor grows large enough to compress the spinal cord, nerve roots, or both, and cause pressure symptoms. The symptoms differ depending on the level from which the tumor arises and is exerting pressure on the spinal cord. Generally, signs and symptoms can include local pain, paraesthesia, radiculopathy, motor weakness, and sphincter control disturbances. In some cases, nonspecific chest or abdominal pain may be the only manifestation of the tumor. In a case series of six patients, those who had thoracic lesions presented with spastic paraparesis [14]. In this case, the patient presented with bilateral lower limb weakness, arising from the left lower limb first, then progressing to the right lower limb in a week. It was associated with numbness starting at the abdomen, which then spread progressively to the lower limbs and body. Upon physical examination, the patient demonstrated reduced sensation below the T6 level, bilateral lower limb hypertonia, bilateral hyper-reflexia of the knee and ankle, and a positive Babinski sign bilaterally.

The Eden classification, which categorizes spinal schwannomas based on their intraspinal and extraspinal involvement, guided our surgical approach for this Type III lesion, characterized by both intraspinal and paraspinal extension where complete resection was achieved through a multidisciplinary strategy combining posterior laminectomy and uniportal VATS [15]. Future prospective studies integrating preoperative functional assessments and long-term follow-up are needed to validate such classification-based approaches and optimize individualized surgical planning for thoracic schwannomas.

Spinal schwannomas are usually asymptomatic, and diagnosis is usually made by an incidental finding or by proper imaging studies once the patient has become symptomatic. Usually, a spinal X-ray and CT scan are used to detect spinal schwannomas, with MRI being considered the gold standard to investigate intradural extramedullary spinal schwannomas. The spinal X-ray and CT scan may reveal an increase in the width of the neural foramen, pedicle erosion, an increased interpedicular distance, and adjacent vertebral body scalloping [16]. In this case, the CT scan of the thoracic spine revealed tumor extension with associated bone remodelling involving the T4 vertebral body, spinous process, right transverse process, lamina, and right pedicle.

Histopathologically, schwannomas classically display Antoni A and/or Antoni B patterns, with nuclear palisades that form Verocay bodies. Immunohistochemistry of the tumor cells typically demonstrates diffuse and strong S100 antigen positivity, with variable CD34, calretinin, TLE1, podoplanin, and SOX2 expression [17]. The Ki-67 proliferative index tends to be low [18]. In this case, the tumor demonstrated all the classical features and showed diffuse S100 positivity, with Ki-67 that ranged from less than 1% to 5%.

Complete surgical excision is the treatment of choice. In this case, it involved a multidisciplinary team of neurosurgical and cardiothoracic surgical teams. If there is no nerve root entrapment, complete excision with no deficits is often achievable. However, if the tumor extensively adheres to nerve roots, incomplete removal is preferred to prevent the risk of postoperative neurological deficits. Complete resection is the main goal to lower the probability of recurrences [13]. However, in cases of spinal schwannomas at any level, complete resection may not be feasible due to adhesion of the tumor to the spinal cord, due to haemorrhage, inflammation, or subpial localization, with the other key factor being other critical structures adhering to the extradural components in the external region of the spinal canal [17]. Postoperatively, the patient's lower limb weakness improved, and she was able to stand and walk without a walking frame at home. Upon physical examination, she demonstrated normal muscle tone, improved power, and no exaggerated or diminished reflexes with a brisk response.

The prognosis of spinal schwannomas is excellent postoperatively, with very little possibility of malignant transformation. The outcome of the surgery itself usually correlates with the condition with which the patient presented preoperatively. Total resection is generally curative if not associated with neurofibromatosis. In contrast, tumors with subtotal resection have a higher rate of recurrence. In an analysis of 40 cases of spinal schwannoma, six were subtotally resected, where two of the six showed recurrence [17].

## Conclusions

Thoracic schwannomas present unique challenges due to their complex anatomical positioning and potential for local tissue invasion. This case highlights the effective management of an IDEM cystic schwannoma using a minimally invasive, multidisciplinary approach combining cardiothoracic and neurosurgical expertise. The use of uniportal VATS facilitated a favourable outcome, demonstrating the technique's utility in addressing paraspinal and pleural lesions while minimising intraoperative and postoperative complications. Future prospective studies are warranted to validate these findings by integrating preoperative functional assessments and long-term follow-up, with the aim of improving surgical risk stratification, individualizing operative planning, and optimizing decision-making in the management of thoracic schwannomas.
